# Impact of different shades of light-emitting diode on fecal microbiota and gut health in broiler chickens

**DOI:** 10.5713/ab.22.0028

**Published:** 2022-04-29

**Authors:** Andrea Ianni, Francesca Bennato, Veronica Di Gianvittorio, Marco Di Domenico, Camillo Martino, Martina Colapietro, Cesare Cammà, Giuseppe Martino

**Affiliations:** 1 Faculty of BioScience and Technology for Food, Agriculture and Environment, University of Teramo, Via Renato Balzarini 1, 64100 Teramo, Italy; 2 Istituto Zooprofilattico Sperimentale dell’Abruzzo e del Molise “G. Caporale”, Via Campo Boario, 64100 Teramo (TE), Italy

**Keywords:** Animal Welfare, Antioxidant Enzymes, Chicken Gut Microbiota, Light-emitting Diode, Matrix Metalloproteinases

## Abstract

**Objective:**

The aim of this study was to characterize the fecal microbiota of broiler chickens reared in the presence of different shades of light-emitting diode (LED) lights, correlating this information with biochemical and molecular evidence that allowed drawing conclusions on the state of health of the animals.

**Methods:**

Overall, the metagenomic approach on fecal samples was associated with evaluations on enzymes involved in the cellular response to oxidative stress: glutathione peroxidase (GPX), superoxide dismutase and catalase; while the inflammatory aspect was studied through the dosage of a proinflammatory cytokine, the interleukin 6 (IL-6), and the evaluation of the matrix metalloproteinases 2 (MMP-2) and 9 (MMP-9). Specifically, analysis was performed on distinct groups of chickens respectively raised in the presence of neutral (K = 3,300 to 3,700), cool (K = 5,500 to 6,000), and warm (K = 3,000 to 2,500) LED lightings, and a direct comparison was performed with animals reared with traditional neon lights.

**Results:**

The metagenomic analysis highlighted the presence of two most abundant bacterial phyla, the Firmicutes and the Bacteroidetes, with the latter characterized by a greater relative abundance (p<0.05) in the group of animals reared with Neutral LED light. The analysis on the enzymes involved in the antioxidant response showed an effect of the LED light, regardless of the applied shade, of reducing the expression of GPX (p<0.01), although this parameter is not correlated to an effective reduction in the tissue amount of the enzyme. Regarding the inflammatory state, no differences associated with IL-6 and MMP-9 were found; however, is noteworthy the significant reduction of MMP-2 activity in tissue samples obtained from animals subjected to illumination with neutral LED light.

**Conclusion:**

This evidence, combined with the metagenomic findings, supports a potential positive effect of neutral LED lighting on animal welfare, although these considerations must be reflected in more targeted biochemical evaluations.

## INTRODUCTION

Chicken meat is one of the most widespread and consumed product of animal origin at the global level. On one hand this is due to the fact that poultry meat represents a cheap protein source, but above all to the fact that these animals are characterized by very short production cycles if compared to other animals of zootechnical interest [1].

In the last decades, several improvements have been introduced in the poultry sector with specific regard to genetics, broiler breast size, fat reduction and above all to the feed efficiency. In fact, feed is the factor that has the greatest impact on the final cost of the product [2]. However, concurrently with the rise in growth rates, the incidence of undesirable conditions also increased. This is the case of metabolic diseases and skeletal deformities that have negative repercussions on the production performances, with non-negligible losses from an economic point of view [3]. Such anomalies have therefore led to the need to identify management strategies that could safeguard and preserve the broiler welfare during the production cycle.

From this point of view, the lighting protocols plays a pivotal role for poultry production and this is the reason for why, especially in recent years, various devices and lighting programs (duration, wavelength, and intensity) have been tested [3,4].

Therefore, the possibility to modify the chicken photoperiod in order to influence the animal health and productivity has gained growing interest over time. In particular, several studies focused their attention to the evaluation of the effects induced by monochromatic light on the welfare and performance of birds.

In poultry sector, several studies have been already conducted on the use of monochromatic light-emitting diode (LED) sources with the purpose of identifying a sustainable strategy from both an environmental and an economic point of view. LED lamps are in fact characterized by high energy efficiency [5], availability of different wavelengths and reduction in rearing costs because of the limited electricity consumption [6].

In the last years, different studies have been conducted on the effect of LED lights application on the production performances and welfare of broiler chickens [7]. In a case study conducted by Hunt [8], the application of LED in a poultry farm was effective in improving the birds’ behaviour, which resulted more calm, with a consequent lower tendency to encounter events of aggression and feather pecking. In addition to this, animals showed able to identify the feed more quickly, with a consequent significant increase in growth performances if compared to chickens reared under conventional lighting protocols. In another study, the birds exposition to a LED lighting program induced a significant improvement of the growth performance in comparison to animals that completed the production cycle in presence of compact fluorescent light. In the same study, was also evaluated the preference of animals for a specific shade of light, finding a higher feed consumption in presence of white LED lighting [9]. The digestion and absorption of the nutrients in animals largely depends by the gut microbiota function and composition; with specific regard to birds this aspect still deserves further studies. However, interesting is the experimentation reported by Li et al [10], in which was evidenced the ability of monochromatic LED (red, blue and white) to affect the growth performance of geese via the gut microbiota, precisely influencing nutrients digestion and absorption.

The recent improvement of 16S rRNA sequencing techniques has made it possible to perform timely and reliable evaluations of the intestinal microbiota, both in humans and animals [11], thus allowing to perform correlations between the type of microbial flora, the intestinal function and the presence of any pathological conditions [12].

Our study therefore takes its cue from this aspect, in an attempt to characterize the gut microbiota of broiler chickens reared in the presence of different shades of LED lighting, correlating this information with biochemical evidence that can allow us to draw conclusions on the state of health of the animals. Specifically, distinct groups of animals were respectively raised in the presence of neutral (K = 3,300 to 3,700), cool (K = 5,500 to 6,000) and warm (K = 3,000 to 2,500) LED lightings, and a direct comparison was performed with animals reared with traditional neon lights. Our hypothesis was that this management strategy could induce positive changes in the fecal microbiota, while improving the chickens’ health. It is in fact for this reason that further analysis were also performed on the intestinal tissue, with the aim of evaluating the presence of inflammatory mediators and the expression of enzymes involved in antioxidant defence: glutathione peroxidase (GPX), superoxide dismutase (SOD) and catalase (CAT). With specific regard to the inflammatory mediators, the attention was focused on the accumulation of inteleukin-6 (IL-6), a pro-inflammatory cytokin, and the activity of zinc-dependent proteases (matrix metalloproteinases 2 [MMP-2] and 9 [MMP-9], also known as gelatinases A and B, respectively) capable of degrading specific structures of the extracellular matrix in the processes of tissue remodeling and repair [13].

## MATERIALS AND METHODS

### Experimental design, animals, farming conditions and sampling

The trial was performed in a commercial poultry farm located in the Abruzzo region (Italy) that in the weeks preceding the experimentation had introduced LED lighting in the breeding boxes. All the procedures regarding the chickens’ management were executed in full agreement with the European regulation dealing with the protection of chickens kept for meat production (European directive 2007/43/EC) [14]. During the trial period, no management practices different from those normally adopted by the company were introduced (rearing protocols normally applied for the production of commercial heavy chickens with live weight equal to 3.20 to 3.50 kg); therefore, was not considered necessary to ask for approval by the ethics committee.

The detailed description of the experimental design has been already reported in a recent publication [15] that was focused on the evaluation of meat quality. Briefly, the study involved a total of 180 broilers (Ross 508; Aviagen Group, Huntsville, AL, USA) that were randomly divided into 4 groups (45 chicks per group). A control group (CTR) was reared in presence of standard neon lights (24.9 lx), and the 3 experimental groups that were respectively reared applying 3 different shades of LED lighting: neutral (NL; K = 3,500 to 3,700; 43 lx), cool (CL; K = 5,500 to 6,000; 48.4 lx) and warm (WL; K = 3,000 to 2,500; 39.6 lx). For each condition have been provided 3 replicates of 15 birds each.

Slaughtering was performed at the end of the normal production cycle, in the commercial abattoir of the company, thus allowing the sampling of the intestinal packets of 30 chickens for each experimental group. The fecal samples were collected at the caecal level immediately after evisceration, while the caecal tract of the intestinal tissue was kept on ice until reaching the laboratory within 60 min from evisceration. For each animal, the intestinal tissue was dissected in order to obtain a portion that was aliquoted and immediately stored at −80°C in anticipation of the biochemical analyzes, while another portion (about 0.80 g), intended for molecular evaluations, was inserted in a vial containing 4 mL of RNALater (Sigma Aldrich, Milan, Italy).

### DNA extraction

DNA extraction was accomplished by means of the Maxwell LEV Blood DNA Kit (Promega, Madison, CA, USA) with specific modifications to the standard protocol as follows: 100 mg of faeces were tranferred to 1.5 mL clean tube and 400 μL Lysis Buffer were added to the samples. The tubes were mixed by vortexing 60 sec and then incubated for 5 min at 95°C. After that, samples were centrifuged at 13,000 rpm for 5 min. We collected 300 μL of supernatant and transfered to new tubes with 30 μL Proteinase K solution followed by a second incubation step at 56°C for 20 min. The total volume was loaded into the cartridges provided by the kit for the final automated step in the Maxwell 16 instrument (Promega, USA). DNA was quantified by Qubit Fluorometer 2.0 (Thermofisher Scientific, Waltham, MA, USA) using the Qubit dsDNA HS (high sensitivity) Assay Kit (Thermofisher Scientific, USA).

### Library prep and sequencing

DNA concentration was normalised by diluting the samples up to 3 ng/μL with pre-polymerase chain reaction (PCR) TE (Tris-EDTA) buffer. Ten μL of input DNA (30 ng) were used for library preparation using the SWIFT AMPLICON 16S+ITS PANEL (Swift Biosciences, Ann Arbor, MI, USA) following the manufacturer’s instructions. The kit provides a single primer pool covering all the variable regions of the 16S rRNA gene (V1-V9) and Illumina-compatible adapters. Sequencing was performed on the MiniSeq (Illumina Inc., San Diego, CA, USA) by the MiniSeq Mid Output Kit (300-cycles) and standard 150 bp paired-end reads. Quality control was performed using FASTQC quality control software v0.11.9.

### Bioinformatic analyses

Bioinformatic processing of raw sequences was performed using the GAIA platform suite (version 2.0). After trimming by trimmomatic (v0.36), Burrows-Wheeler Alignment tool (version 0.7.13) was used to map the high-quality reads/pairs against custom-made databases created by Sequentia Biotech including National Center for Biotechnology Information (NCBI) sequences. Reads were clustered into operational taxonomic units (OTUs) using an in-house Lowest Common Ancestor algorithm. Minimum identity thresholds were applied to classify reads into strains, species, genus, family, order, class, phylum, and domain levels. OTU distribution among samples was used to calculate rarefaction curves. Alpha diversity was calculated using Phyloseq (version 3.11). Dissimilarities between pairs of samples were estimated using the Bray–Curtis dissimilarity index. The relative abundances of OTUs were expressed in terms of percentage of reads while differential abundance analysis was performed by DESeq2 package (version 3.11) using negative binomial generalized linear models [16].

### Enzyme-linked immunosorbent assay of interleukin-6, glutathione peroxidase, superoxide dismutase and catalase

Commercial kits (MyBioSource, Inc., San Diego, CA, USA) were used in order to determine the amount of IL-6, GPX, and CAT, and the activity of SOD in extracts of caecal intestinal tissue. For the preparation of the extracts, 1 g of tissue was weighed and then minced to small pieces which were homogenized in phosphate-buffered saline with a glass homogenizer on ice. To further break the cells, samples were sonicated and then subjected to 2 cycles of freeze/freeze-thaw. The homogenates were then centrifugated at 5,000×g for 5 minutes at 4°C, and supernatants were collected.

The assays were performed following the manufacturer’s instructions and the results were reported in ng/L (IL-6), ng/mL (GPX), U/mL (SOD) and pg/mL (CAT).

### Total RNA extraction, cDNA synthesis and analysis of polymerase chain reaction products

Total RNA was extracted from chicken instestinal tissue and conserved in RNA later, by using a Quick-RNA MiniPrep plus kit (Zymo Research, Irvine, CA, USA) following the procedure described by the manufacturer. The concentrations of RNA samples were then quantified by using the NanoDrop 2000 Spectrophotometer (ThermoFisher, USA).

cDNA were synthesized by exploiting the Wonder RT-cDNA Synthesis Kit (Euroclone, Milan, Italy) and amplified by polymerase chain reaction (PCR) in presence of specific primers (Table 1) for SOD, CAT, and β-actin was used as house-keeping gene. The PCR mixture was composed by 1.5 U of a recombinant thermostable DNA polymerase (Wonder Taq Hot Start; Euroclone, Italy), 0.4 μM of each primers and a reaction buffer containing 5 mM dNTPs, 15 mM MgCl_2_, stabilizers and enhancers. The amplification was obtained by applying the following thermal program: 95°C for 1 min followed by 30 cycles at 95°C for 15 sec, Tm (melting temperatures reported in Table 1) for 15 sec, 72°C for 30 sec with a final extension at 72°C for 7 min. The PCR products were resolved on 1% agarose gel and the quantitative analysis of visualized spots was performed by using the Gel Doc 2000 software (Bio Rad Laboratories, Hercules, CA, USA).

### Zymographyc analysis of matrix metalloproteinases 2 and 9

The zymographyc evaluation of gelatinases (MMP-2 and MMP-9) was performed on tissue extracts previously obtained for the enzyme-linked immunosorbent assay evaluations. Volumes of each sample corresponding to 15 μg of total proteins were diluted in a non-reducing sample buffer without heating and resolved by 8% sodium dodecyl sulfate-polyacrylamide gel electrophoresis (SDS-PAGE) containing 0.15 mg/mL type B gelatin (Sigma Aldrich, Italy). The gels were then incubated for 45 min in a renaturation buffer (50 mM Tris-HCl pH 8.0, containing 2.5% Triton X-100) to remove SDS. Subsequently, a 24 h incubation in the developing buffer (50 mM Tris-HCl pH 8.0, containing 5 mM CaCl2, 200 mM NaCl and 0.02% Brij 35) was performed to allow enzyme renaturation. Gels were then stained in a 0.1% solution of Coomassie Blue R250 in 40% (v/v) methanol and 10% (v/v) acetic acid, and the intensity of the corresponding bands was determined in the same way previoulsy described for the agarose gel electrophoresis.

### Statystical analysis

The results are expressed as mean value±standard deviation. The analysis of variance was performed by applying the One-way analysis of variance, and significantly different groups were ranked using the post hoc comparison test (Tukey test) at 95% (p<0.05) confidence level. The data were analyzed statistically with the SigmaPlot 12.0 software package (Systat Software Inc., San Jose, CA, USA) for Windows operating systems.

## RESULTS

### Characterization of gut microbiota composition

**Figure 1:**
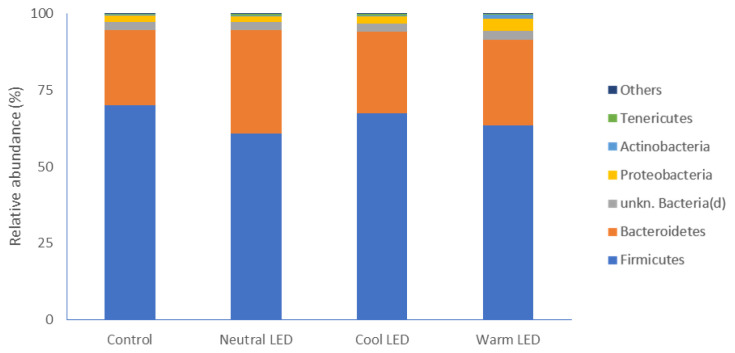
Most represented phyla of bacteria and relative abundances observed in fecal samples obtained from chickens reared in the presence of neutral (K = 3,300 to 3,700), cool (K = 5,500 to 6,000) and warm (K = 3,000 to 2,500) LED lightings; a direct comparison was performed with animals reared with traditional neon lights (control). LED, light-emitting diode.

The evaluation of gut microbiota allowed to identify different phyla. As reported in Figure 1, regardless of the lighting protocol, the most represented phylum in all samples was that of Firmicutes without significant differences between the various groups involved in the experimentation. Below, in descending order of abundance, Bacteroidetes were found to have relatively higher abundance values in the groups reared in the presence of LED lights, although the data is significant compared to the control only in the case of chickens subjected to Neutral LED lighting (p<0.05). Less abundant phyla were represented by Proteobacteria, Actinobacteria, and Tenericutes, without significant differences between groups (Figure 1).

**Figure 2:**
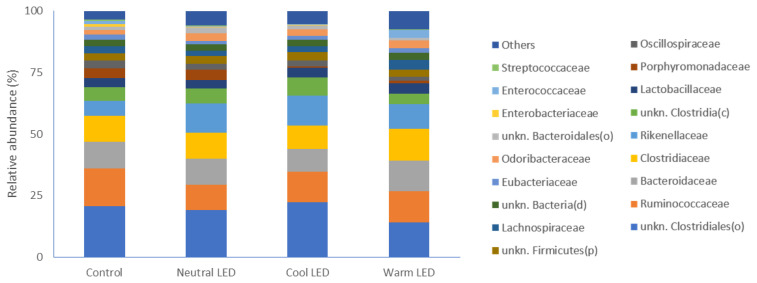
Most represented families of bacteria and relative abundances observed in fecal samples obtained from chickens reared in the presence of neutral (K = 3,300 to 3,700), cool (K = 5,500 to 6,000) and warm (K = 3,000 to 2,500) LED lightings; a direct comparison was performed with animals reared with traditional neon lights (control). LED, light-emitting diode.

With specific regard to bacterial families, the most represent ed were in the order: unknown Clostridiales, Ruminococcaceae, Bacteroidaceae, Clostridiaceae, Rikenellaceae and unknown Clostridia (Figure 2) with no significant differences between the groups under evaluation.

No index used to describe the α-diversity has showed significant variations between the groups (p>0.05) (Table 1).

### Expression of antioxidant enzymes (GPX, SOD, and CAT) in gut tissue

**Figure 3:**
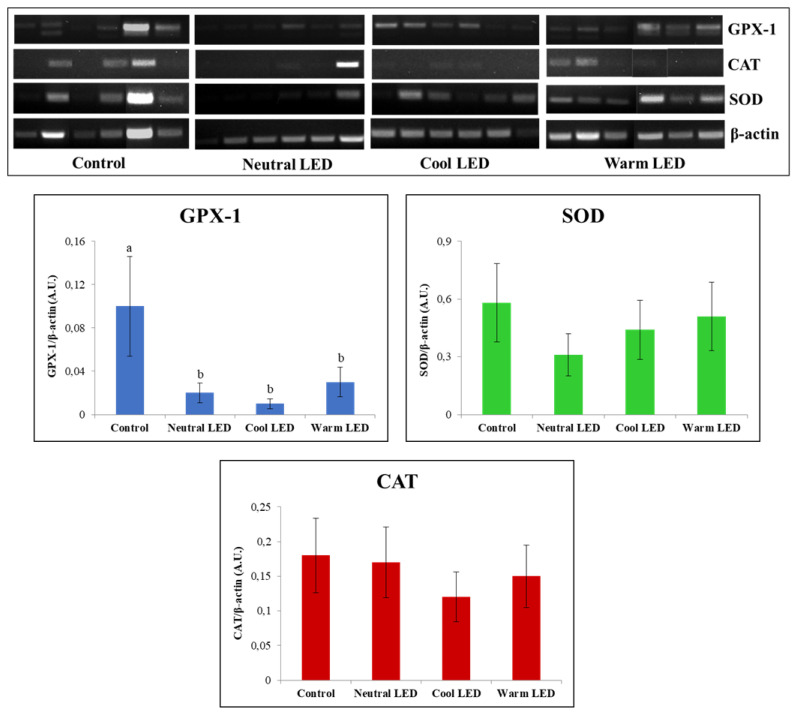
Reverse-transcription polymerase chain reaction (RT-PCR) analysis of interleukin-6 (IL-6), glutathione peroxidase (GPX), superoxide dismutase (SOD) and catalase (CAT) on intestinal tissue samples obtained from chickens reared in the presence of neutral (K = 3,300 to 3,700), cool (K = 5,500 to 6,000) and warm (K = 3,000 to 2,500) LED lightings; a direct comparison was performed with animals reared with traditional neon lights (control). LED, light-emitting diode. ^a,b^ Means with different letters are significantly different (p<0.05).

The reverse transcription (RT-PCR) was useful for the characterization of gene expression associated to GPX, SOD, and CAT in gut tissue. The obtained results (Figure 3) showed an overlapping pattern of expression between the various groups as regards SOD and CAT (p>0.05) while the evaluation performed on GPX highlighted a reduced relative abundance of transcripts (p<0.05) in the intestinal tissue obtained from animals reared in the presence of LED lighting. From this point of view, it should be also emphasized that no significant statistical differences were observed between neutral, cool, and warm LED lighting (p>0.05).

### Evaluation of the inflammatory state: the accumulation of IL-6 and the MMP activity

In Table 2 are reported the results concerning the IL-6 dosage in gut tissue. As can be seen, no significant differences (p>0.05) were found between the various groups subjected to analysis.

The zymographic approach was effective in highlighting the specific activity of MMP-2 and MMP-9, also allowing to discriminate in both cases between proenzymatic and active enzymatic forms.

**Figure 4:**
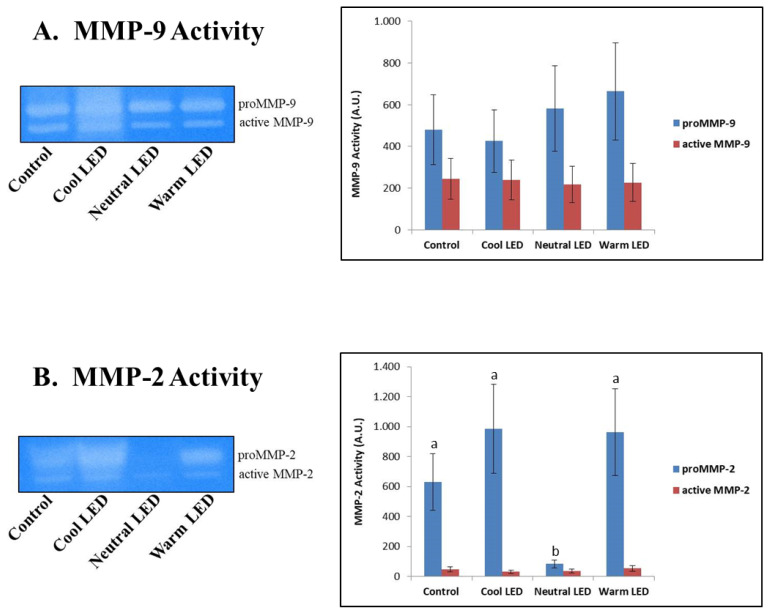
Zymographic evaluation of matrix metalloproteinases 2 (MMP-2) and 9 (MMP-9) activities in extracts of intestinal tissue obtained from chickens reared in the presence of neutral (K = 3,300 to 3,700), cool (K = 5,500 to 6,000) and warm (K = 3,000 to 2,500) LED lightings; a direct comparison was performed with animals reared with traditional neon lights (control). The Gelatin zymograpy shown in the figure is representative of the overall data. LED, light-emitting diode. ^a,b^ Means with different letters are significantly different (p<0.05).

The results reported in Figure 4 did not show any difference on the activity of MMP-9, both as regards the zymogen and the active form of the enzyme. On the other hand, in the case of MMP-2 was found a significant reduction in the activity associated with the proenzymatic form in the Neutral LED group compared to all the other experimental groups (p<0.01); in the same samples, however, no variations were associated with the degree of activity of the active enzymatic form (p>0.05).

## DISCUSSION

The use of fecal material for the characterization of the gut microbiota in chickens represents undoubtedly the most suitable strategy [17].

Our attention was specifically focused on the caecal intestinal portion. The gut microbiota of chickens, at the level of the caecal tract, is in fact considerably more complex than other intestinal segments. Specifically, it has been estimated that the fecal digestate in this anatomical portion has a microbial population composed of approximately 1,000 different species, with an absolute count close to 10^10^ CFU [18]. The major phyla that we have identified in all experimental groups are represented by gram-positive Firmicutes and gram-negative Bacteroidetes, a finding in full agreement with what has been reported in several studies in which the poultry intestinal microbiome has been characterized [19]. In addition to this, it must be highlighted that the specific presence of this two dominant phyla in poultry gut has been related with a significant increase in the weight gain [20]. From this point of view, the most interesting data of our study concerns Bacteroidetes, which, in the case of animals reared in the presence of LED lighting, have higher relative mean values compared to that of the control group, a difference which is however significant only in the treatment with neutral LED light. The main importance of this phylum, which comprises bacteria that vary from strict aerobes to obligate anaerobes, is mostly associated with the degradation of nutrients that reach the cecum, favoring their absorption. In particular, members of the Bacteroidetes phylum are reported to express and release different isoforms of glycoside hydrolases and polysaccharide lyases that are crucial for their contribution to the increase in nutrients availability in the gut [21].

In addition to this, it must be also reported that the four genera belonging to the phylum of Bacteroidetes (*Bacteroides*, *Prevotella*, *Porphyromonas*, and *Flavobacterium)* also include some potential pathogens for birds [22], or bacteria capable of promoting the engraftment of overt pathogens in the intestine. An example could be represented by *Bacteroides thetaiotaomicron* (Bt) that is resident in the attachment sites of enterohemorrhagic *Escherichia coli* (EHEC). Specifically, Bt was demonstrated to be able to increase the virulence potential of EHEC through the release of the transcription factor Cra, whose expression and function is strongly influenced by the concentration of specific sugars in the surrounding microenvironment [24]. In addition to this, should be also reported that both Firmicutes and Bacteriodetes have been identified as major players in the short-chain fatty acid metabolism. Specifically, Firmicutes are involved in the production of butyrate and propionate, whereas propionate alone represents the main metabolic product of Bacteroidetes [2]. Donohoe et al [25] reported butyrate to provide approximately the 70% of the energy used by normal colonic epithelial cells; furthermore, was also evidenced his ability to increase the thickness of the mucus layer, contributing to the prevention of the invasion by pathogenic bacteria. The observed increase in Bacteroidetes, especially in the group reared with neutral LED light, could therefore have contributed to a reduction in butyrate levels, with potentially negative repercussions on the functionality of the intestinal mucosa. However, the inflammation markers considered in this study did not show significant variations between the various groups. First of all, in the intestinal tissue was evaluated the amount of IL-6; this cytokine, together with IL-1 and tumour necrosis factor-α, represents an indicator of macrophage activity as a consequence of viral and bacterial infections [26]. Furthermore, IL-6 was also reported to represent a general index responsive to acute stress in poultry. Specific studies have highlighted the ability of illumination to influence the levels of IL-6 and other cytokines, as an effect of changes in circulating melatonin levels [27]. This assumption therefore supports the hypothesis that experimental lighting programs, based on the use of different shades of LED light, did not induce variations in the synthesis and release of this hormone compared to the group of animals raised with standard lighting.

The zymographic analysis of gelatinases (MMP-9 and MMP-2) made it possible to highlight the activity of these enzymes at the level of the caecal intestinal tissue. In mammals, the increase in the expression and activity of these enzymes is commonly associated with both acute and chronic inflammatory events, as well as with tumor invasion events [28]; with close regard to our study, it is of particular interest the fact that these enzymes, especially in recent years, received particular attention also in the characterization of pathologies that afflict poultry on farm [29]. In our study, MMP-9 (gelatinase B) did not show significant differences between the various experimental groups, while aroused interest data concerning the activity of MMP-2 in the group reared with Neutral LED light. In this case was highlighted only minimal activity by the zymogen, namely the inactive form of the enzyme. The explanation for this finding could lie in a reduced expression of the gene that codes for the enzyme and certainly not in a greater conversion of the pro-enzymatic form into the active form, since the activity of the latter is absolutely comparable to that found in the other experimental groups. Another explanation could instead be found in the fact that in the “neutral LED” group the inactive enzyme was secreted more rapidly into the extracellular environment [30], thus reducing its function at the cytosolic level. A study performed by Ganguly et al [31], addressed the topic concerning the relationship between melatonin and gelatinases’ function. Specifically, authors investigated the effect of melatonin on the regulation of MMP-2 and MMP-9 in a rat model suffering from indomethacin-induced gastric ulcer. Interestingly, the results showed melatonin be able to prevent gastric ulceration by reducing the expression and secretion of pro-MMP-2 in a dose-dependent manner. What has just been said could justify, at least in part, a potential role of the neutral LED light in inducing a greater release of melatonin, although specific evaluations must be carried out to verify this hypothesis.

The evaluations performed on the enzymes of the antioxidant response (GPX, SOD, and CAT) were always conducted with a view to implementing the amount of information about the health of the gut, since these enzymes are able to counteract the deleterious action exerted by reactive oxygen species produced by aerobic metabolism, such as hydrogen peroxide, superoxide anions and hydroxyl radicals [32]. In the specific case of this study, we assumed that variations in chicken gut microbiome have significant effects on the expression and function of these enzymes. A specific example is given by the study conducted by Bai et al [33] in which it was shown that feeding broilers with the probiotic *Bacillus subtilis* was effective in increasing the mRNA expression levels of both GPX and glutathione. The results obtained from our evaluations highlighted fairly consistent findings regarding the amount of transcript produced and the enzyme actually present in the cytosolic environment. In particular, the most noteworthy data concerns the significant reduction in the expression of GPX-1 in all animals reared in the presence of LED light, regardless of the shade applied. However, this reduction in the quantity of transcripts did not correspond to a significant reduction in the amount of the protein, although the calculated quantities showed in all cases average values that tend to be lower than the control. These evaluations have therefore highlighted a picture difficult to decipher, above all due to the fact that it goes against what has already been reported in other studies in which was reported the effect of different shades of LED light on the antioxidant system of chickens. An example is given by the recent study conducted by Seyidoğlu et al [34] who evaluated the serum enzyme activities of GPX, SOD and CAT in Ross 308 broiler chicks exposed for the 42 days of the trial to white (control), red, green and blue LED light. This experimentation has shown the ability of green light to significantly increase all antioxidant activities; in any case, in none of the experimental groups were highlighted values of enzymatic activity lower than those calculated in the control group. At this point, in an attempt to explain the data acquired in our study, could be advanced a hypothesis on the fact that there is not necessarily a direct correlation between the amount of the enzyme present and its specific activity, due to the fact that even minimal changes in the cellular microenvironment can vary the kinetic parameters of the enzyme produced. Obviously, this point deserves more in-depth evaluations to remove speculative conclusions from the field.

## Figures and Tables

**Table 1:** α-Diversity measured by observed operational taxonomic units (OTUs), Chao1, Shannon, Simpson and Fisher indexes

Item	Observed OTUs	Chao1	Shannon	Simpson	Fisher
Control	677.67±15.07	961.52±15.70	3.90±0.08	0.95±0.01	100.76±2.72
Neutral LED	727.33±34.07	1,087.45±24.59	3.88±0.04	0.95±0.01	104.74±4.52
Cool LED	669.67±95.64	988.47±126.39	3.80±0.11	0.94±0.01	99.99±10.74
Warm LED	821.50±9.19	1,191.48±43.56	3.52±0.33	0.95±0.01	88.11±29.47

LED, light-emitting diode.

Indexes were calculated in fecal samples obtained from chickens reared in the presence of neutral (K = 3,300 to 3,700), cool (K = 5,500 to 6,000) and warm (K = 3,000 to 2,500) LED lightings; a direct comparison was performed with animals reared with traditional neon lights (control).

Differences between the groups were not significant (p>0.05).

**Table 2:** ELISA of IL-6, GPX, SOD and CAT on intestinal tissue samples obtained from chickens reared in the presence of neutral (K = 3,300 to 3,700), cool (K = 5,500 to 6,000) and warm (K = 3,000 to 2,500) LED lightings; a direct comparison was performed with animals reared with traditional neon lights (control)

Item	IL-6 (ng/L)	GPX (ng/mL)	SOD (U/mL)	CAT (pg/mL)
Control	11.83±2.44	5.23±0.67	47.42±4.85	392.52±6.78
Neutral LED	13.31±3.65	5.12±0.61	48.49±4.48	385.19±5.74
Cool LED	14.37±3.26	5.06±0.62	50.30±4.80	394.33±6.11
Warm LED	12.16±3.45	5.14±0.24	47.62±4.90	378.04±5.50

ELISA, enzyme-linked immunosorbent assay; IL-6, interleukin-6; GPX, glutathione peroxidase; SOD, superoxide dismutase; CAT, catalase; LED, light-emitting diode.

Differences between the groups were not significant (p>0.05).
